# Case Report: Endovascular thrombectomy for acute ischemic stroke with bilateral internal carotid artery fibromuscular dysplasia

**DOI:** 10.3389/fcvm.2025.1583321

**Published:** 2025-08-07

**Authors:** Zhi Zheng, Chen Yang, Xufeng Meng, Li Yang, Yunfei Hao

**Affiliations:** ^1^ The First School of Clinical Medicine, Gansu University of Chinese Medicine, Lanzhou, China; ^2^ Department of Cerebrovascular Diseases, Gansu Provincial Hospital, Lanzhou, China

**Keywords:** fibromuscular dysplasia, bilateral internal carotid artery, internal carotid artery dissecting aneurysm, acute cerebral infarction, endovascular thrombectomy, digital subtraction angiography

## Abstract

**Background:**

Fibromuscular Dysplasia (FMD) is a rare, idiopathic, non-inflammatory, and non-atherosclerotic disease that often presents with stenosis of medium or small arteries, potentially leading to acute cerebral infarction. Due to its atypical symptoms, FMD is prone to being missed or misdiagnosed, and the optimal treatment strategy for patients with acute cerebral infarction accompanied by FMD remains unclear.

**Case description:**

A 41-year-old male patient presented to the emergency department with right-sided limb weakness and slurred speech for 4 h. He had a history of gout and smoking. Physical examination revealed grade 2 muscle strength in the right limb and a NIHSS score of 15. Head CT showed an acute cerebral infarction in the left temporal and parietal lobes. Cerebral angiography revealed “string-of-beads” stenosis of the bilateral internal carotid arteries (multifocal fibromuscular dysplasia), occlusion of the left internal carotid artery (ICA) at its origin (TICI grade 0), and a dissecting aneurysm in the C1 segment. After comprehensive assessment, the patient was diagnosed with ICA fibromuscular dysplasia and underwent emergency endovascular mechanical thrombectomy. Intraoperatively, tirofiban was used for anti-thrombotic therapy, and postoperative management included aspirin and statin therapy for secondary prevention. Follow-up cerebral angiography at 1 and 3 months showed patency of the left ICA, with a Modified Rankin Scale (mRS) score of 1, and no new ischemic events.

**Conclusion:**

FMD is a rare non-atherosclerotic disease, with cerebral vessel involvement being relatively common and presenting a variety of clinical symptoms, which poses challenges in diagnosis and treatment. For patients with FMD accompanied by acute vascular occlusion, endovascular mechanical thrombectomy is an effective treatment option, and in terms of treatment strategy, combining anti-platelet therapy can effectively improve neurological function and achieve a favorable prognosis.

## 1 Introduction

Fibromuscular Dysplasia is a rare idiopathic, segmental, non-inflammatory, and non-atherosclerotic disease characterized by abnormal cell proliferation and arterial wall distortion. It most commonly affects the renal arteries, followed by the internal carotid arteries, but can occur in nearly any artery in the body (1, 2). Data on FMD mainly come from international registries like the European International FMD Registry and Initiative (FEIRI), but its prevalence in the general population is still unknown (3). Symptomatic carotid FMD has a reported prevalence of around 0.2% (4), and a 25-year consecutive autopsy series at the Mayo Clinic showed a cerebral vascular FMD prevalence of 0.02% (5). Significant variations exist in the clinical and angiographic features of FMD patients across different populations. Most reports indicate that FMD is more common in females, while male patients are relatively rare and more prone to aneurysms and arterial dissections (6, 7). However, a recent study found that in the Asian population, there is a higher proportion of male patients, who tend to be diagnosed at a younger age, have a higher proportion of focal FMD, and a lower incidence of multifocal vascular diseases, aneurysms, and dissections (8).

In the First International Consensus on the Diagnosis and Management of Fibromuscular Dysplasia, based on angiographic findings, FMD is categorized into focal and multifocal types (2). Multifocal FMD, accounting for about 90% (9), is characterized by alternating stenosis and dilatation, creating a “string-of-beads” appearance, and is commonly seen in the mid and distal segments of arteries (10). Focal FMD, about 10% (11), is marked by single, concentric or tubular stenosis and can occur anywhere in the artery (6). A majority of patients are asymptomatic (12). Symptomatic patients mainly present with chronic headaches, pulsatile tinnitus, or symptoms of neurological complications, including transient ischemic attacks, ischemic strokes, carotid artery dissections, subarachnoid hemorrhages, and ruptures of unruptured aneurysms (13–16). Note that only 15%–20% of FMD patients have arterial dissections and 20%–25% have aneurysms (17). Dissections or aneurysms alone aren't sufficient for FMD diagnosis (10). Although DSA is the gold standard for diagnosing FMD (2), most medical centers prefer computed tomography angiography (CTA) or contrast-enhanced magnetic resonance angiography (MRA) for initial diagnosis. This is because patients with FMD have a higher risk of iatrogenic dissection or stenosis during DSA procedures (18). Doppler ultrasound can be used as an adjunctive tool to detect turbulent flow and curvatures within the internal carotid artery (2). This report details a case of bilateral internal carotid artery FMD in a male patient to enhance diagnostic and management strategies for this complex vascular disease.

## 2 Case presentation

A 41-year-old male presented to our emergency department with right-sided limb weakness and dysarthria of 4 h duration. The patient presented with sudden-onset right-sided limb weakness, dysarthria, and diaphoresis without any obvious precipitating factors 4 h prior to the initial medical evaluation. The absence of nausea, vomiting, visual blurring, and other associated symptoms helped differentiate the condition from brainstem stroke and hemorrhagic stroke. According to the family, the patient's responses were vague following the onset of symptoms. Emergency cranial computed tomography (CT) imaging revealed an acute cerebrovascular event, and the patient was subsequently admitted to the neurology department for treatment. The patient has a history of gout but denies any history of hypertension, diabetes, hyperlipidemia, or stroke. He has a smoking history, and the family history is non-contributory.

**Figure 1:**
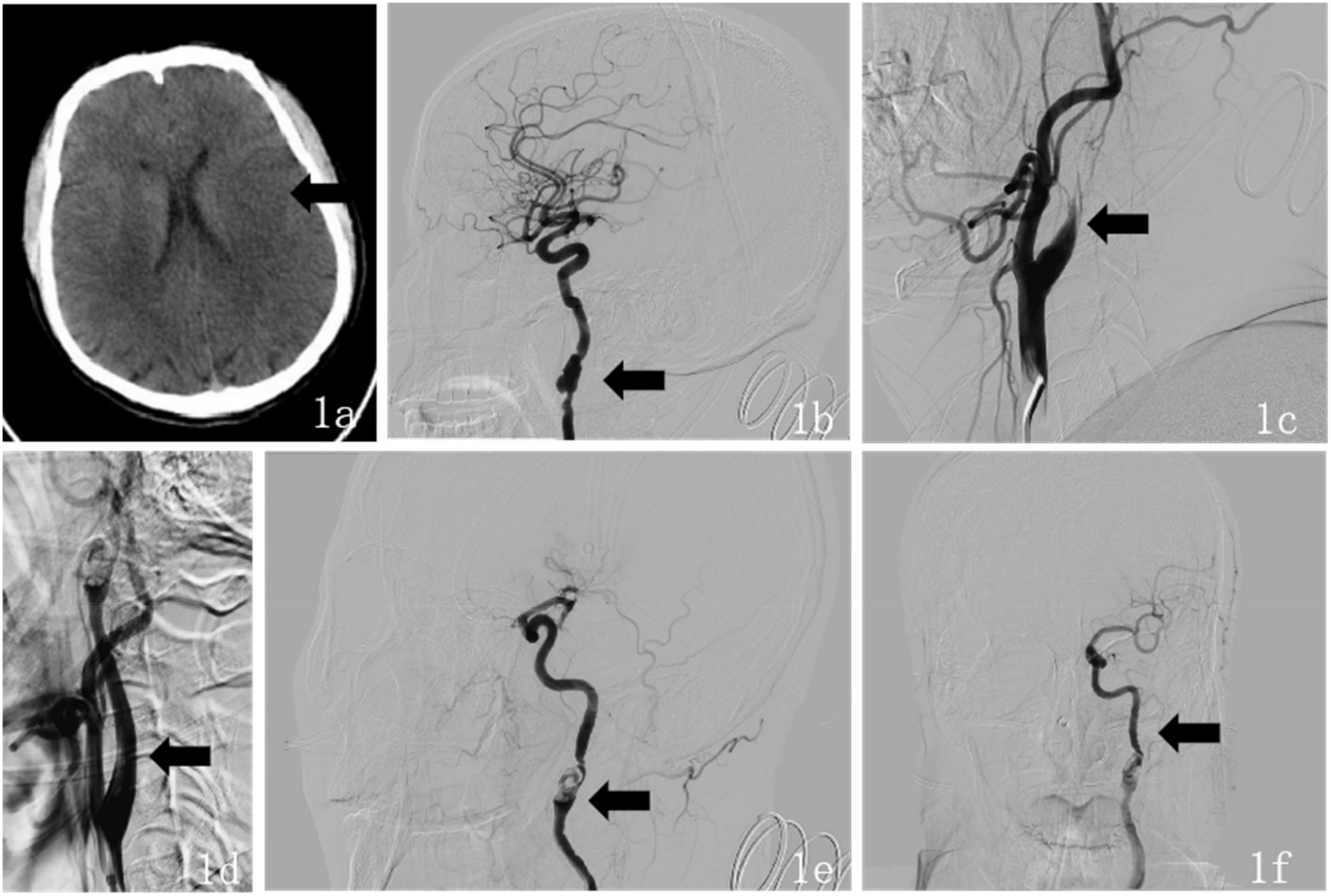
**(a)** Preoperative (October 17, 2024) head CT findings: axial CT shows a large area of slightly low-density lesions in the left temporal-parietal lobe. **(b)** Intraoperative DSA findings during endovascular therapy: The lateral DSA demonstrates a “string of beads” appearance in the right ICA, accompanied by a dissecting aneurysm in the C1 segment. **(c)** Lateral DSA shows occlusion at the origin of the left ICA. **(d)** Intraoperative lateral DSA shows recanalization of the left ICA. **(e)** Intraoperative lateral DSA revealed areas of alternating stenosis and dilatation in the left ICA, with a dissecting aneurysm in the C1 segment. **(f)** Intraoperative anteroposterior DSA shows “string of beads” changes in the left ICA at the C1 segment, with restored distal blood flow.

Upon admission, the patient had a temperature of 36.8 °C, heart rate of 75 beats per min, respiratory rate of 18 breaths per min, and blood pressure of 125/75 mmHg. The patient exhibited clouded consciousness, dysarthria, and irrelevant responses to questions. Examination revealed intact facial sensation bilaterally, with a shallow right nasolabial fold, accompanied by dysarthria and hoarseness. The tongue deviated to the right, and muscle strength in the right limbs was graded 2/5, with normal muscle tone and negative bilateral Babinski signs. The National Institutes of Health Stroke Scale (NIHSS) score was 15 (consciousness: 4, facial palsy: 1, right upper limb: 3, right lower limb: 3, language: 2, dysarthria: 2). The mRS score was 4 at the time of initial medical assessment, compared to a pre-stroke mRS score of 0. Laboratory tests showed uric acid 474.00 µmol/L, magnesium 2.33 mmol/L, and bicarbonate 16.20 mmol/L. Thromboelastogram revealed reaction time (R) of 1.0 min, coagulation time (K) of 0.0 min, angle of 26.3 degrees, maximum amplitude (MA) of 9.9 mm, coagulation index (CI) of −5.2 min, and G of 0.5 d/sc. No significant abnormalities were found in the complete blood count, coagulation profile, or immune function tests. Emergency cranial CT revealed a patchy, slightly hypodense area in the left temporoparietal lobe (Figure 1a), suggesting an acute-phase cerebral infarction. Subsequently, at 02:30 digital subtraction angiography (DSA) was performed using the Sellinger technique with right femoral artery puncture, placement of a 5F arterial sheath, and systemic heparinization. A 5F curved catheter was used for aortic arch and full-brain angiography. The DSA revealed a Type III aortic arch, “string of beads” appearance in the right internal carotid artery, and a dissection aneurysm in the C1 segment (Figure 1b). Additionally, the left internal carotid artery showed occlusion at its origin, with a modified Thrombolysis in Cerebral Infarction (mTICI) grade 0 (Figure 1c), and generalized cerebral vascular tortuosity and sclerosis. DSA demonstrated segmental, string-of-beads changes with local dilatation and eccentric stenosis in the extracranial right ICA, accompanied by a dissection aneurysm but without evident wall plaque, consistent with the imaging features of FMD. Considering the absence of fever, elevated inflammatory markers, anemia, non-tubular stenosis, clinical manifestations of hereditary connective tissue diseases associated with aneurysms and dissections, and a family history of aneurysms or dissections, combined with evidence of FMD, the condition was differentiated from large-vessel vasculitis, spontaneous artery dissection, and hereditary connective tissue diseases. Based on a comprehensive evaluation of preoperative imaging and the patient's clinical history, the final diagnosis was internal carotid artery fibromuscular dysplasia and left ICA occlusion.

After providing a detailed explanation of the patient's condition and treatment plan to the family, they agreed to proceed with intra-arterial endovascular thrombectomy. During the procedure, intravenous dexmedetomidine was continuously infused. The 5F arterial sheath was subsequently replaced with an 8F sheath, and the 8F guide catheter was positioned at the origin of the left ICA. A 6F intracranial support catheter was successfully navigated to the occlusion site, where negative pressure aspiration was performed. After the support catheter was removed, a significant amount of thrombus was successfully extracted. Immediate angiography revealed patency of the left ICA (Figure 1d), with alternating stenosis and dilatation in the left ICA and a dissection aneurysm in the C1 segment (Figure 1e). Tirofiban 10 ml was slowly injected into the artery, and a continuous intravenous infusion was maintained. After 15 min of observation, follow-up angiography showed restoration of distal blood flow with a mTICI grade 3 (Figure 1f). The procedure was successfully completed, and the patient's vital signs remained stable throughout the surgery. Postoperatively, the sheath was removed, a closure device was applied to seal the puncture site, and the wound was compressed for 15 min with no active bleeding. Finally, the wound was dressed with a sterile bandage, and the patient was safely transported back to the Intensive Care Unit (ICU).

**Figure 2:**
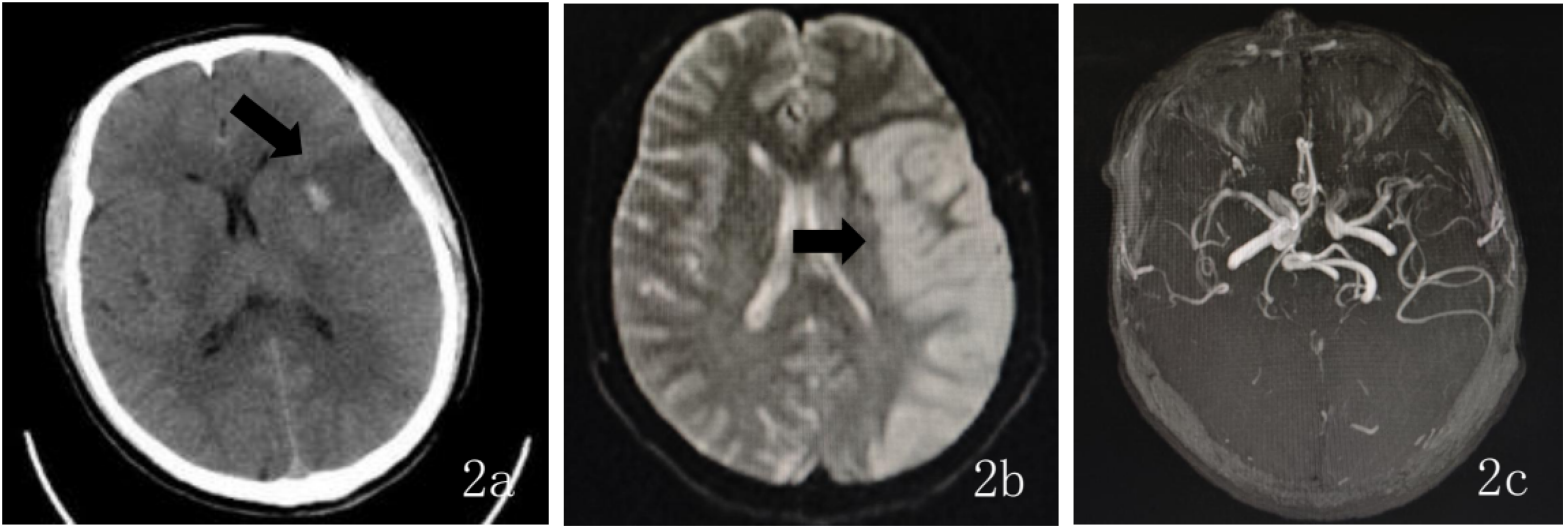
**(a)** Postoperative (October 17, 2024) head CT findings: axial CT shows a large area of slightly low-density lesions in the left frontal, temporal, and parietal lobes, and a small area of slightly high-density lesions in the left basal ganglia. **(b)** Postoperative (October 20, 2024) MR head imaging, including plain scan, diffusion-weighted imaging (DWI): Axial DWI shows high signal intensity in the left temporal, parietal, and insular lobes. **(c)** Postoperative (October 20, 2024) magnetic resonance angiography (MRA) finding: MRA shows no significant stenosis or occlusion of the intracranial major arteries.

Postoperative neurological examination revealed no new neurological deficit signs. Follow-up cranial CT showed acute cerebral infarction in the left frontal, temporal, and parietal lobes, and a small amount of new hemorrhage in the infarcted margin (Figure 2a). The punctate hemorrhage at the infarct edge likely resulted from hemorrhagic transformation following reperfusion in the ischemic area post-acute stroke. This is associated with secondary hemorrhage caused by endovascular mechanical thrombectomy. After assessing the patient's hemorrhagic transformation and hemorrhage risk, the patient was administered oral enteric-coated aspirin 100 mg once daily, rosuvastatin calcium 10 mg once daily, 3-n-butylphthalide for improved circulation, urinary hallucinogens (a tissue kallikrein that improves hemodynamics and exerts neuroprotective effects by dilating blood vessels, promoting collateral circulation formation, and inducing angiogenesis) for neuroprotection, and supportive treatment. On the second postoperative day, the patient exhibited mixed aphasia, and muscle strength in the right limbs improved to grade 3. On the third postoperative day, routine follow-up MR brain scan, diffusion-weighted imaging, and MRA showed: (1) large-area acute/subacute cerebral infarction in the left frontal, parietal, and temporal lobes and left insular cortex; (2) a cavity infarction in the right temporal lobe; and (3) no significant abnormalities in brain MRA (Figures 2b,c). The right temporal lobe lacunar infarction was consistent with the first postoperative CT imaging and not a new infarction. On the fourth postoperative day, the patient's right-limb strength improved to grade 4, and he was able to walk with assistance. Though mixed aphasia persisted, it presented as non-fluent aphasia with severe deficits in auditory comprehension, naming, reading, and writing, but with relatively preserved repetition. Reexamination of head CT showed that the density of the infarcted area in the left frontotemporal and parietal lobes further decreased, and the amount of bleeding in the infarct margin area decreased. The patient was hospitalized for 8 days and discharged with clear consciousness, stable vital signs, and fair mental status. Upon discharge, the patient continued oral enteric-coated aspirin 100 mg once daily and rosuvastatin calcium 20 mg once daily. At the 1-month follow-up, DSA showed a patent left ICA and FMD in the C1 segment of both internal carotid arteries, consistent with prior angiography findings for FMD. At the 3-month outpatient follow-up, the patient was in good general condition with recovered speech function. After active rehabilitation exercises for limb mobility, he could walk unassisted and perform basic daily activities independently, which effectively alleviated the concerns of both the patient and his family. His mRS score was 1, muscle strength in the right limbs had improved to grade 4, and no new ischemic symptoms were observed. And Time line was shown in Table 1.

**Table 1:** Timeline.

Date	Event
October 17, 1:57 am	Admitted for sudden right-sided limb weakness and dysarthria of 4 h duration.
	Emergency head CT: a patchy, slightly hypodense area in the left temporoparietal lobe (Figure 1a).
October 17, 2:30 am	DSA: Type III aortic arch, “string of beads” appearance in right ICA, C1 segment dissection aneurysm (Figure 1b). Left ICA origin occlusion, mTICI grade 0 (Figure 1c).
	Following informed consent, Endovascular Thrombectomy was performed.
	Post-procedure DSA: Left ICA patency (Figure 1d), string-of-beads changes, C1 segment dissection aneurysm (Figure 1e), distal blood flow restored, mTICI grade 3 (Figure 1f)
October 17, 4:30 am	Post-op vitals stable; transferred to ICU.
October 17, 10:48 am	Head CT: acute infarction in left frontotemporal and parietal lobes with minor hemorrhagic transformation (Figure 2a).
October 19	Mixed aphasia, right-sided muscle strength grade 3.
October 20	Brain MRI: large infarction in left cerebral hemisphere. MRA normal (Figures 2b,c). Right imb muscle strength grade 4, walks with assistance; mixed aphasia persists.
October 21	Right limb muscle strength grade 4, walks with assistance; mixed aphasia persists.
	Follow-up Head CT: the amount of bleeding in the infarct margin area decreased
October 24	Discharged with mixed aphasia and right-sided muscle strength grade 4; rehab ongoing.
1 month post-discharge	DSA: left ICA patency, FMD in bilateral ICA C1 segments.
3 months post-discharge	Speech function restored; ongoing rehabilitation; independent in basic activities.

## 3 Discussion

FMD is a rare, idiopathic, segmental, non-inflammatory, and non-atherosclerotic vascular disease characterized by abnormal cell proliferation and distortion of the arterial wall structure. The disease commonly affects the renal arteries and internal carotid arteries (1, 2). The prevalence of FMD affecting the ICA remains unclear. Most patients are female, although male patients are more prone to developing aneurysms and arterial dissections (3, 19). The reported average age of onset ranges from 43 to 52 years (6, 7). The exact etiology of FMD is still debated, but it is believed to be associated with genetic factors, hormonal levels, and smoking, all of which may contribute to the progression of the disease (2, 13, 20). Few familial FMD cases have been reported. However, FMD is considered to have a complex genetic basis. For instance, the recurrent pathogenic variant c.1540G>A in collagen type V alpha-1 gene (COL5A1) has been implicated as potentially associated with multifocal FMD (21). This patient was a 41-year-old male with no history of common risk factors such as hypertension or atherosclerosis. He presented with symptoms of ischemic stroke, such as limb weakness and slurred speech. However, because the patient exhibited mixed aphasia during the disease course, common manifestations such as headache and pulsatile tinnitus were not detected on physical examination. Emergency DSA was performed to investigate the intracranial vascular cause, revealing alternating areas of stenosis and dilation in the bilateral ICA C1 segments, where the dilated segments were larger than the normal arterial lumen. We found that the findings were consistent with the common sites and typical angiographic features of multifocal FMD (10), and initially suspected multifocal FMD of the internal carotid artery. This can be differentiated from the single, concentric (length <1 cm) or tubular (length ≥1 cm) smooth stenosis seen in focal FMD (6). Furthermore, this condition must be distinguished from atherosclerosis, which typically does not affect this vascular region (2). Imaging also revealed a carotid artery dissection aneurysm. Due to the difficulty in obtaining clinical specimens and the effectiveness of imaging studies in diagnosis, the patient did not undergo a biopsy. The patient had no atherosclerotic risk factors, family history of cerebrovascular disease or special diseases, normal inflammatory markers, and no systemic non-specific inflammatory manifestations. DSA showed a “string of beads” appearance in the extracranial segments of both internal carotid arteries, along with a dissection aneurysm. Since the stenosis was not caused by other conditions like atherosclerosis or vasculitis, a diagnosis of bilateral internal carotid artery FMD was made.

Currently, there is a lack of large-scale clinical trials for patients with FMD, and treatment decisions are primarily based on expert consensus and case reports. For asymptomatic patients, regardless of the degree of stenosis, surgical or endovascular interventions are generally not recommended, although regular imaging follow-up is advised (2). In the absence of cerebrovascular-related conditions, initiating prophylactic antiplatelet therapy to prevent thrombosis formation is considered reasonable (2, 22). For patients presenting with concomitant cerebrovascular disease, therapeutic management parallels that of non-FMD patients (22, 23). In cases of large vessel occlusion or severe carotid artery stenosis, urgent interventions such as intravenous thrombolysis, endovascular thrombectomy, and carotid artery stenting may be considered (24, 25). Additionally, for secondary prevention of stroke, it is recommended that FMD patients with ischemic cerebrovascular events be treated with anticoagulants or antiplatelet medications, such as aspirin, for a duration of at least 3–6 months (22, 24). Moreover, statin therapy is generally not recommended in patients without concomitant atherosclerosis (26, 27). Furthermore, smoking patients demonstrate a higher incidence of adverse events compared to non-smoking individuals, and patients exhibiting smoking behavior should be counseled to quit smoking and establish healthy lifestyle modifications (20, 28). Management followed the same principles applied to non-FMD cases. In this patient with bilateral internal carotid artery FMD and severe left ICA occlusion, urgent endovascular thrombectomy was performed after medical evaluation, successfully restoring blood flow. However, bilateral carotid artery dissection aneurysms persisted. The patient presented with a dual diagnosis of left internal carotid artery occlusion and an aneurysm, which posed a therapeutic challenge. Despite the risk of rupture, intervention with advanced endovascular techniques, along with preventive management of complications, was deemed proactive and necessary. Recent studies indicate that the risk of intraoperative aneurysm rupture is 5.8%, but endovascular thrombectomy continues to be an effective and generally safe treatment option (29). Postoperatively, the patient's consciousness and right-sided limb strength improved significantly; however, the speech impairment showed limited improvement, likely due to the infarction being located in the language-related functional area. In accordance with the secondary prevention recommendations for stroke, the patient was administered aspirin at a dosage of 100 mg once daily to prevent recurrent stroke, and it was recommended that the patient undergo lifelong antiplatelet therapy. Additionally, smoking may increase the prevalence of major vascular events (20). The patient was informed of this risk and agreed to quit smoking. This male patient has a higher risk of carotid artery dissection and should avoid activities that may cause neck strain (30). He has bilateral carotid artery dissection aneurysms at the C1 segment of the internal carotid arteries, which have a high rupture risk. However, there is currently insufficient high-quality evidence to support either conservative or interventional treatment for patients with unruptured intracranial aneurysms, and given that the patient is in the acute phase of ischemic stroke, immediate interventional treatment is not recommended (31). Regular follow-up and observation are chosen, with a planned re-evaluation in the future to determine whether to perform aneurysm embolization (32). Following endovascular therapy, the patient showed significant improvement in neurological symptoms, including consciousness disturbances and limb weakness. Subsequent follow-ups indicated good functional recovery and enhanced quality of life, reflecting the efficacy of endovascular treatment for FMD with ischemic stroke, and indicating a favorable prognosis.

In summary, male patients with FMD are more susceptible to severe complications such as aneurysms and strokes, which increases the complexity of diagnosis and treatment. This case report clearly outlines the imaging diagnostic criteria, treatment methods, and prognosis. Given the high risk in young male patients, Endovascular Thrombectomy was employed as a critical intervention. The report further confirms the efficacy of Endovascular Thrombectomy in FMD-related stroke and offers important reference and new diagnostic and therapeutic insights for the limited literature on FMD-related stroke in young male patients.

## 4 Conclusion

In conclusion, bilateral ICA FMD is a rare vascular disease, with frequently leading to cardiovascular and cerebrovascular diseases and leading to a variety of clinical symptoms. The condition demonstrates significantly lower prevalence in the male and is more likely to be associated with arterial dissection or aneurysms. DSA is the gold standard for diagnosing this disease, and when combined with CTA/MRA, it can effectively identify the characteristic “string of beads” stenosis and arterial dissection. For patients with acute vascular occlusion, this study demonstrates the successful use of emergency endovascular thrombectomy to restore vessel patency, confirming the therapeutic value of this technique in acute large vessel occlusion associated with FMD. Furthermore, a postoperative individualized treatment strategy combining antiplatelet therapy and statins helps to improve patient outcomes. This case underscores the importance of increasing awareness of FMD in clinical practice and highlights the need for early vascular imaging evaluation in patients with unexplained stroke to avoid delays in diagnosis and treatment.

## Data Availability

The raw data supporting the conclusions of this article will be made available by the authors, without undue reservation.
